# Author Correction: Zebra finches identify individuals using vocal signatures unique to each call type

**DOI:** 10.1038/s41467-020-16398-z

**Published:** 2020-06-04

**Authors:** Julie E. Elie, Frédéric E. Theunissen

**Affiliations:** 1 Helen Wills Neuroscience Institute, UC Berkeley, Berkeley, CA 94720 USA; 2 Department of Psychology, UC Berkeley, Berkeley, CA 94720 USA

**Keywords:** Signal processing, Animal behaviour

Correction to: *Nature Communications* 10.1038/s41467-018-06394-9, published online 02 October 2018.

The original version of this article contained errors in the deposition locations of data and code.

The original Data availability statement read:

“The custom Python code used to calculate the acoustical features is available as the BioSound class in sound.py found in https://github/theunissenlab/soundsig. Tutorials with example data are found in https://github/theunissenlab/BioSoundTutorials. The complete library of vocalizations is available at: https://drive.google.com/drive/folders/1etvuPjaNHV9oFPgUAuLxP3bk1aWfj3Pl?usp=sharing. The behavioral data and behavioral analysis code are available from the corresponding authors upon reasonable request.”

The correct version replaces this with:

“The custom Python code used to calculate the acoustical features is available as the BioSound class in sound.py found in https://github.com/theunissenlab/soundsig. Tutorials with example data are found in https://github.com/theunissenlab/BioSoundTutorial. The complete library of vocalizations is available at Figshare: 10.6084/m9.figshare.11905533.v1. The behavioral data and behavioral analysis code are available from the corresponding authors upon reasonable request.”

The original version also included the incorrect Github URLs in the first paragraph of the ‘Acoustical analysis’ section of the Methods:

“All acoustical features were obtained using custom Python code from the Theunissen lab (BioSound class in sound.py found in https://github/theunissenlab/soundsig; BioSound Tutorials with examples are found in https://github/theunissenlab/BioSoundTutorials).”

The correct version replaces this with:

“All acoustical features were obtained using custom Python code from the Theunissen lab (BioSound class in sound.py found in https://github.com/theunissenlab/soundsig; BioSound Tutorials with examples are found in https://github.com/theunissenlab/BioSoundTutorial).”

These have been corrected in both the PDF and HTML versions of the Article.

