# Supplementary material for: Zebra finches identify individuals using vocal signatures unique to each call type
Source: Nat Commun. 2018 Oct 2;9:4026. doi: 10.1038/s41467-018-06394-9 (PMC6168511; doi:10.1038/s41467-018-06394-9)
Supplement: Supplementary file 1 — Supplementary Information [file 41467_2018_6394_MOESM1_ESM.pdf]

Zebra finches identify individuals using vocal signatures unique to each call type

Elie & Theunissen

Supplementary Figures:

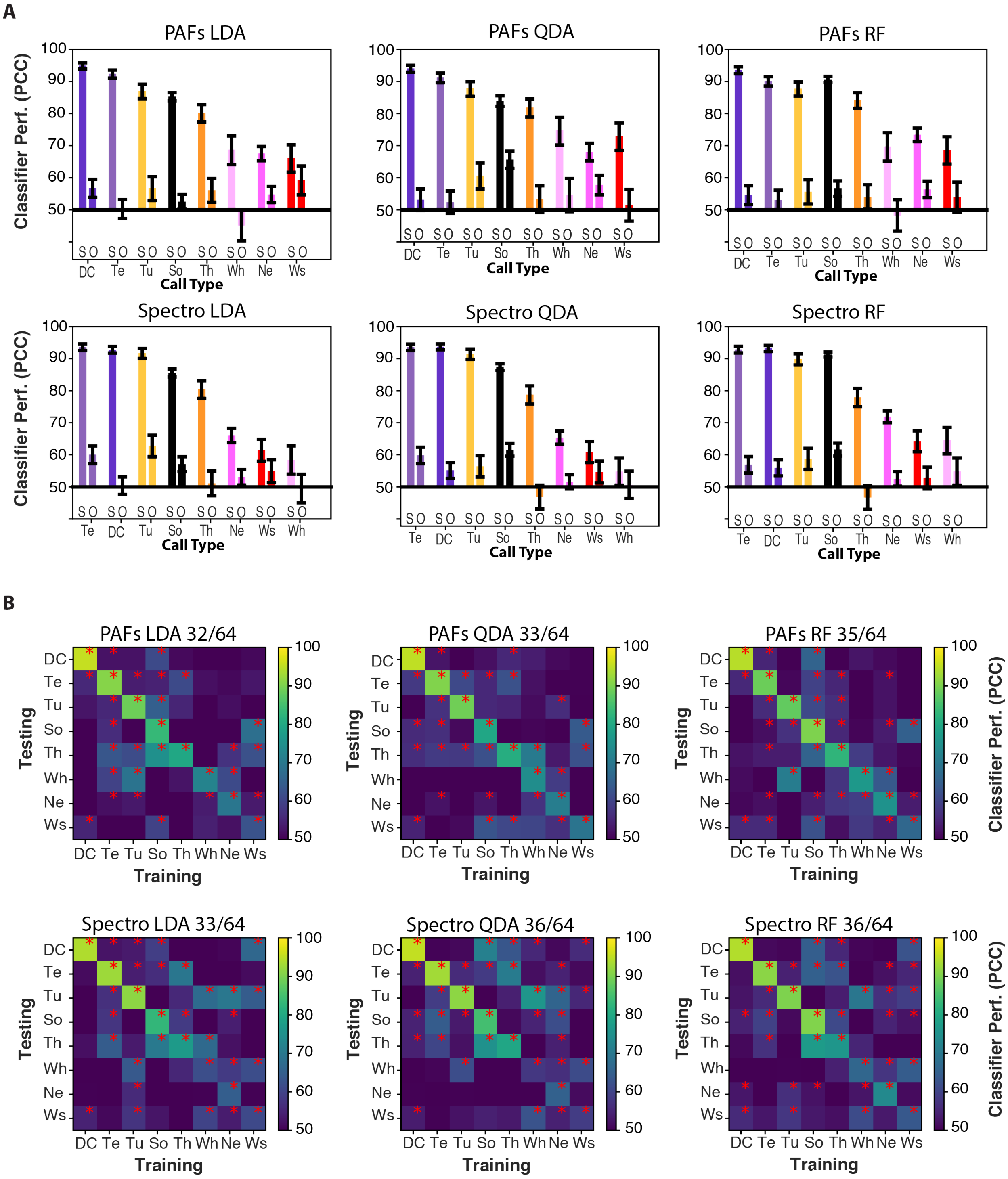

Supplementary Fig. 1.

Effect of feature space and type of classifier on the generalization of the acoustic parameters used for vocalizer classification across call types. These panels complement Figs.

4 and 6 and compare the performance (Percent of correct classification, PCC) of three different regularized classifiers (Linear Discriminant Analysis, LDA; Quadratic Discriminant Analysis, QDA; Random Forest, RF) and two feature spaces (18 pre-defined acoustic features, PAFs; spectrogram, Spectro) for testing the existence of voice-cues. The performance of the classifiers is always quantified by cross-validation, separating training and testing data. For the *Same* condition in A and the diagonal in B, the testing dataset is composed of other renditions of the same call type. For the *Other* condition in A and the off-diagonal in B, the testing dataset is composed of call renditions from other call types. If the same acoustic features are used to classify vocalizers irrespective of call type, then the performance of a given classifier should be similar between *Same* and *Other*. A. Classifiers' performance at categorizing vocalizers when tested on each call type (DC, Te, Tu, So, Th, Wh, Ne, Ws; labels are defined in Figure 4A) and trained either with vocalizations of the *Same* call type (S) or with vocalizations of all *Other* call types (O). Error bars indicate 95% confidence intervals. Even if the performance drastically drops for every vocalization type between the *Same* and *Other* conditions, several performance values stay above chance level indicating some degree of transferability of the acoustic features learned to discriminate vocalizers from all other categories. Changing the features representing the vocalizations or the type of classifier used does not drastically change this result. B. Performance of classifiers on pairwise sets of call types in the two different feature spaces. The color code indicates the classifier performance when trained with the call types indicated in columns and tested on the call type indicated in rows. Red stars indicate significance of the PCC compared to chance level (direct binomial test, $p<0.05$). Irrespective of the feature space or the classifier, the performance of classification drops when the training and testing sets are not from the same call type (bins outside of the diagonal).

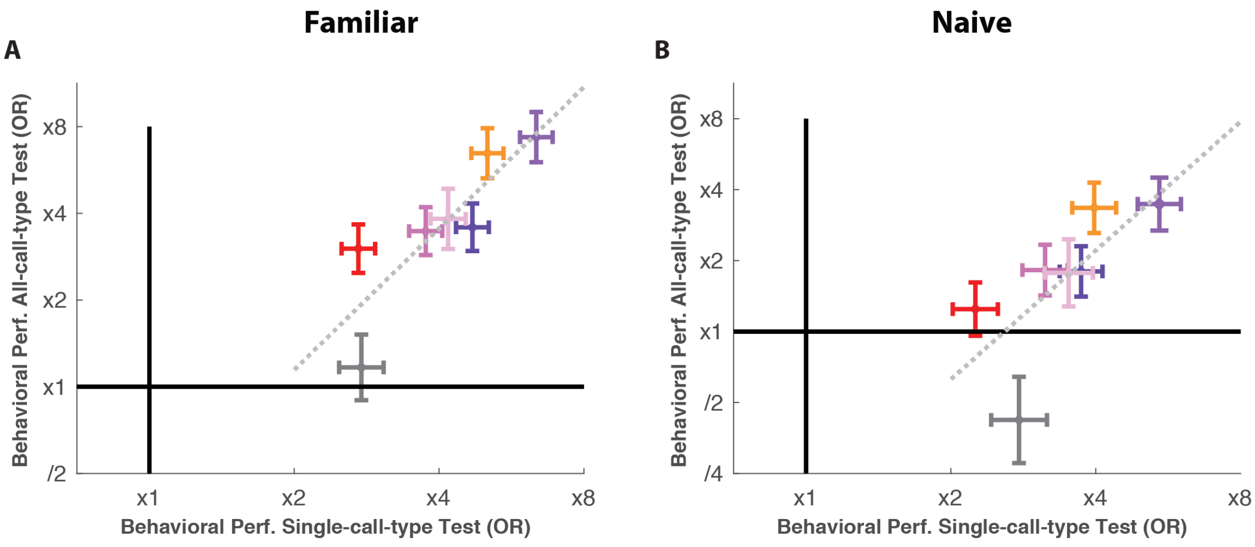

Supplementary Fig. 2.

Correlation of the behavioral performance between single-call-type tests and all-call-type tests when subjects became familiar to the vocalizers during previous single-call-type tests (A, familiar, n=13, two-tailed Pearson test, r = 0.8511, $t_{(5)}$ = 3.625, p=0.0151) or when subjects were not exposed to the same vocalizers (B, naive, n=7, two tailed Pearson test, r = 0.7173, $t_{(5)}$=2.302, p=0.0696). To account for the random effect of subjects, behavioral performance as measured by the odds ratio (OR) is given as the estimates of the GLME coefficients and their 95% confidence intervals, with VocType and CallType:VocType as fixed effects and Subject as random effect. The grey lines depict the linear regression line between the two variables.

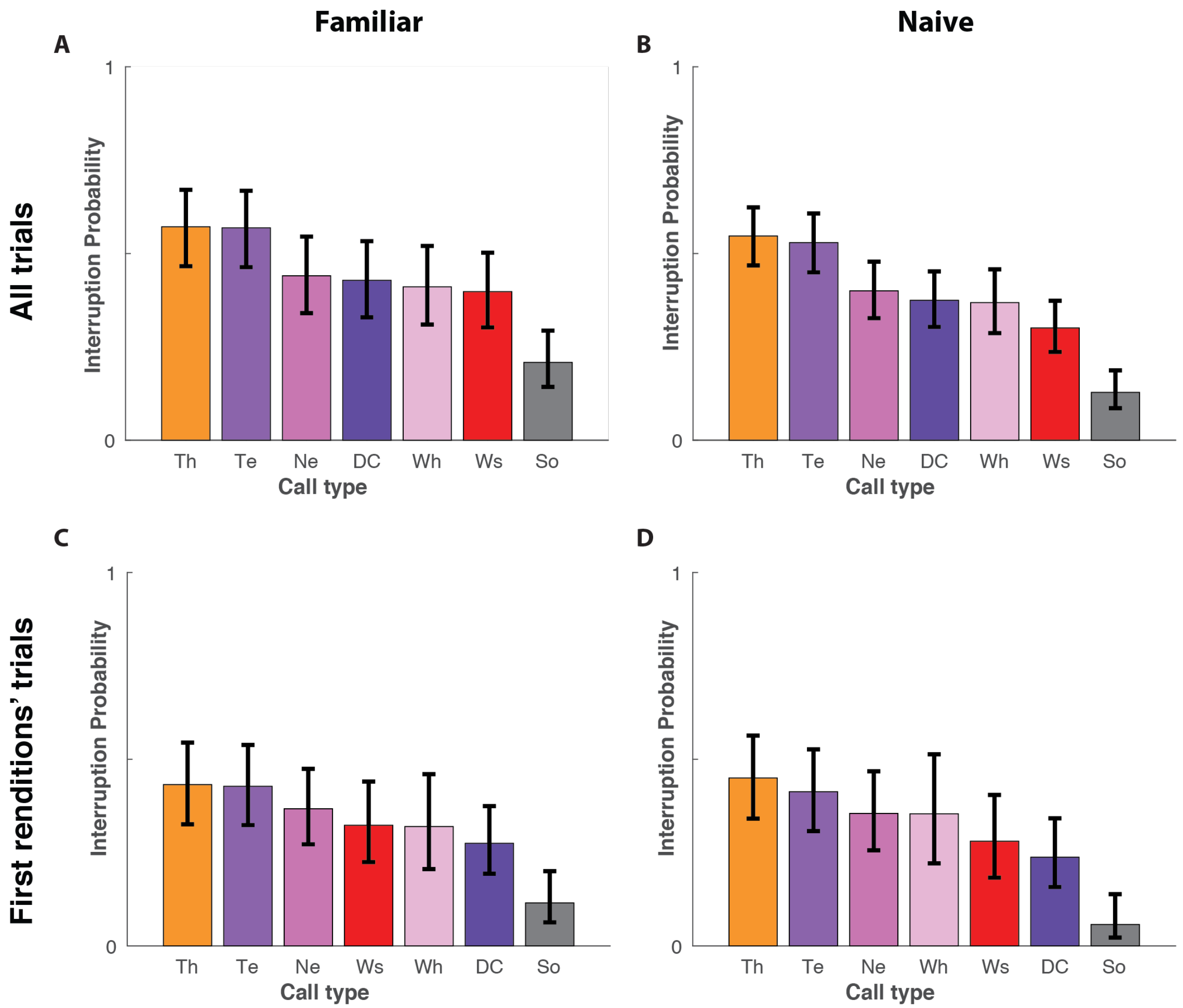

Supplementary Fig. 3.

Effect of call type on the probability of interruption in all-call-type tests. The four bar plots illustrate the probability of interruption of each call type in the all-call-type tests performed by the subjects who got familiar to the vocalizers during previous single-call-type tests (n=13, A and C) or naive to the vocalizations of the vocalizers (n=7, B and D). To account for the random effect of subjects, the values of probabilities are obtained from the coefficients of a GLME predicting the probability of interruption with CallType, VocType and VocType:CallType as fixed effects and Subject as a random effect. Error bars correspond to the 95% confidence intervals. The GLME is run either on the full data set (A, χ^2_6=48.9, p=7.8904x10^{-9} and B χ^2_6=45.2, p=4.319x10^{-8}) or on a set restricted to vocalization renditions heard once or twice (C, χ^2_6=17.2, p=0.0084594; D, χ^2_6=17.2, p=0.0086757).

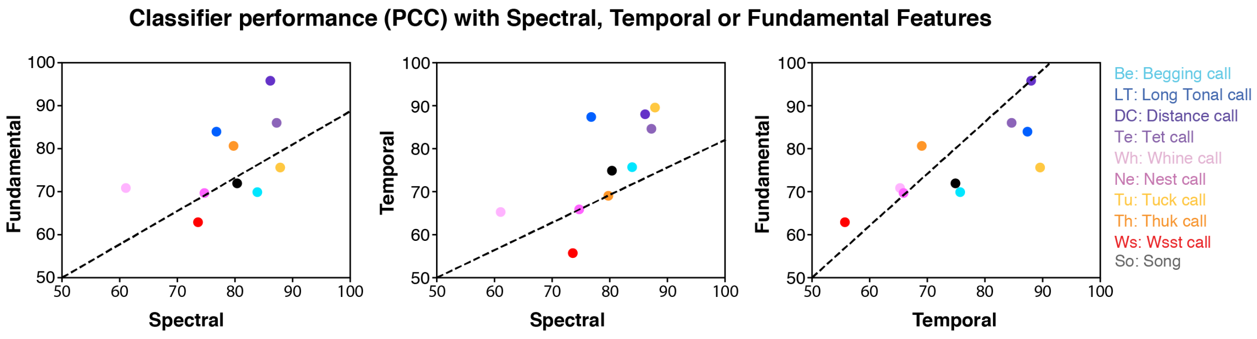

Supplementary Fig. 4.

Increase role of temporal and fundamental features in coding identity information as compared to call type information. This figure complements Figure 8C and shows scatter plots of the classifier performance as measured by the percent of correct classification (PCC) at categorizing pairs of vocalizers within each call type when trained and tested in the space defined by 3 sets of PAFs: Spect, 8 spectral parameters only; Temp, 5 temporal parameters only; Fund, 5 fundamental parameters only. The dashed lines indicate the relative performance of the classifier at discriminating call type (Call Type classification) when using the same set of PAFs. Dots above the dashed line indicate call types for which the relative contribution of the set of PAFs on the y-axis vs the set of PAFs on the x-axis for identity discrimination is larger as compared to Call Type classification. For example, in the first two scatter plots, contact calls (DC, LT and Te) are all above the dashed lines. This indicates that the relative contribution of Temporal and Fundamental features as compared to Spectral features is larger for encoding identity information than it is for encoding call type information.

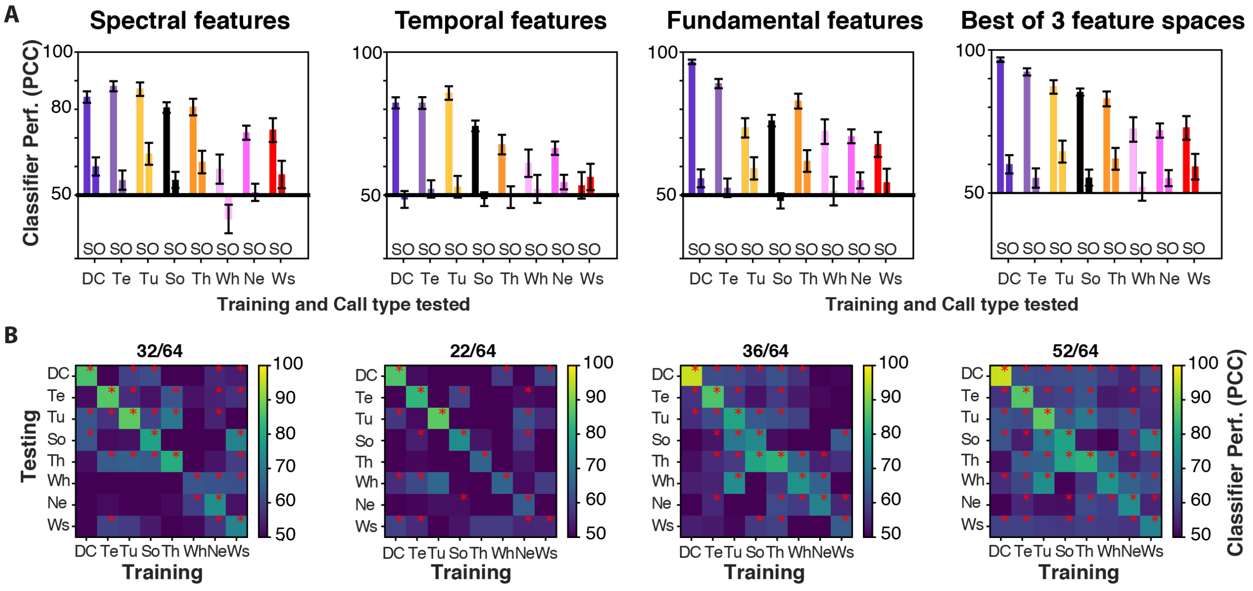

Supplementary Fig. 5.

Generalization of spectral, temporal and fundamental parameters used for vocalizer classification across call types. These figure panels complement Figure 8 and investigate which type of acoustic features are most shared across call types akin to voice-cues. The PAFs are grouped as spectral, temporal or fundamental features and the performance of the regularized LDA classifier is measured when it is trained on different call types than the one it is tested on. If the same type of acoustic feature is used to classify vocalizers irrespective of call types, then the performance of the classifier should stay high for that set of features in this generalization test. **(A) Classifiers' performance (as measured by PCC) at categorizing vocalizers when tested on each call type** (DC, Te, Tu, So, Th, Wh, Ne, Ws) and trained either with vocalizations of the *Same* call type (S) or with vocalizations of all *Other* call types (O). Error bars indicate 95% confidence intervals. Note that even if the performance drastically drops for every call type and for all 3 sets of features when the training switch from *Same* to *Other*, performance values stay above chance level for Spectral and Fundamental features, indicating that these features act as voice-cues to some extent. **(B) Performance of the classifier on pairwise sets of call type for the three sets of PAFs**. The color code indicates the classifier performance (PCC) when trained with vocalizations from the call type indicated in columns and tested on vocalizations from the call type indicated in rows. Red stars indicate significance of the PCC compared to chance level as revealed by a direct binomial test ($p<0.05$). Irrespective of the set of PAFs, the performance of classification drops when the training and testing sets are not from the same call type (bins outside of the diagonal). Note that this is particularly true for temporal features.

Supplementary Table:

Supplementary Table 1.

Log likelihood ratio tests performed on generalized linear mixed effect models. The variable indicated in bold in the Wilkinson formula correspond to the tested variable. The *figure* column indicates where the data are presented (Text, main text; SF, Supplemental figure). Nobs, number of observations; Ngrp, number of random categories; χ, Chi-square; df, degrees of freedom. The names, value and standard error of the coefficients in full model are given in the last column. Predicted variables: PCC, percent of correct classification according to vocalizer ID by the regularized Linear Discriminant Analysis (LDA); PCCsem, percent of correct classification according to call type by the regularized LDA; Int, Number of interrupted stimuli in behavioral tests; CR, number of correct responses in behavioral tests (interruption of NoRe stimuli, non-interruption of Re stimuli). Fixed effects: CallType: call type; Feature: acoustic feature type (spectral, temporal of fundamental); RendRank, presentation rank of each rendition during behavioral tests; Session: session number in the behavioral tests; TrainType: type of training for the classifier (same call type or other call types); VocRank, presentation rank of vocalizations from each vocalizer (Re or NoRe) during behavioral tests ; VocType, vocalizer type (Re or NoRe). Random effects: VocPair, pair of vocalizers; Subject, subject ID; Date, date of the behavioral test.

Wilkinson Formula and **tested** fixed-effect	Figure	Nobs	Ngrp	χ	df	p-value	Coefficients of model in units of Probability		
							name	value	SE
**LDA Data**									
Pcc ~ **CallType** + (1|VocPair)	4A	933	424	4406.5	9	$<2.2\times10^{-16}$	CallType_Ws	0.62092	0.02015
							CallType_Be	0.85266	0.00878
							CallType_DC	0.95988	0.00245
							CallType_LT	0.92636	0.00852
							CallType_Ne	0.68722	0.00986
							CallType_So	0.83746	0.00666
							CallType_Te	0.91659	0.00389
							CallType_Th	0.81646	0.01235
							CallType_Tu	0.89720	0.00844
							CallType_Wh	0.69032	0.02027
Pcc ~ **TrainType** + CallType + (1|VocPair)	8A	402	38	6399.7	9	$< 2.2\times10^{-16}$	TrainType Same	0.86209	0.00603
							TrainType_Other	0.54601	0.01160
Pcc ~ **Feature** + CallType +(1|VocPair)	8C	2799	424	853.76	2	$<2.2\times10^{-16}$	Feature_Spect	0.83347	0.00326
							Feature_Temp	0.78987	0.00281
							Feature_Fund	0.79501	0.00332
PccSem ~ **Feature**	8C	546	0	13.894	2	0.0009614	Feature_Spect	0.62637	0.03492
								0.43406	0.03576

acoustical features are averaged per bird across all renditions for a particular category							Feature_Temp<br>Feature_Fund	0.50549	0.03606
							Coefficients of model in Linear Response units		
							name	value	SE
**Single-call-type tests data**									
Int ~ **VocType** + (1|Subject)	4B	1118	13	2814.2	1	0	Intercept	-1.4542	0.14791
							VocType_NoRe	1.4203	0.028852
Int ~ VocType + **VocType:CallType + ** (1|Subject)	4B	1118	13	700.71	8	0	Intercept	-1.4524	0.14641
							VocType_NoRe	0.99982	0.040892
							CallType_Be: VocType_NoRe	-0.085331	0.054141
							CallType_DC: VocType_NoRe	0.54597	0.042854
							CallType_LT: VocType_NoRe	0.56831	0.051087
							CallType_Ne: VocType_NoRe	032245	0.043543
							CallType_So: VocType_NoRe	0.014672	0.056603
							CallType_Te: VocType_NoRe	0.85154	0.042881
							CallType_Th: VocType_NoRe	0.61772	0.042478
							CallType_Wh: VocType_NoRe	0.42955	0.045781
Int ~ VocType + VocType:CallType + **VocType:Session +** (1|Subject)	5A	1118	13	308.72	2	0	Intercept	-1.4518	0.14521
							VocType_NoRe	0.73492	0.045307
							CallType_Be: VocType_NoRe	-0.090301	0.054429
							CallType_DC: VocType_NoRe	0.55467	0.043032
							CallType_LT: VocType_NoRe	0.56071	0.051331
							CallType_Ne: VocType_NoRe	0.33104	0.043755
							CallType_So: VocType_NoRe	-0.014539	0.056975
							CallType_Te: VocType_NoRe	0.85984	0.043053
							CallType_Th: VocType_NoRe	0.62351	0.042708
							CallType_Wh: VocType_NoRe	0.42873	0.045978
							VocType_NoRe:Session_2	0.20305	0.029119
							VocType_NoRe:Session_3	0.48738	0.02853
Int ~ **VocType** + (1|Subject)<br>(Dataset restricted to Session1)	5A	363	13	270.95	1	0	Intercept	-1.0982	0.1306
							VocType_NoRe	0.83231	0.052428
Int ~ **VocType** + (1|Subject)<br>(Dataset restricted to renditions heard once or twice)	5B	429	13	15.804	1	7.0264×10^{-5}	Intercept	-1.2622	0.14219
							VocType_NoRe	0.41178	0.10368

Int ~ VocType + **VocType :CallType** + (1|Subject)<br>(Dataset restricted to renditions heard once or twice)	5B	429	13	18.421	8	0.018283	Intercept<br>VocType_NoRe<br>CallType_Be: VocType_NoRe<br>CallType_DC: VocType_NoRe<br>CallType_LT: VocType_NoRe<br>CallType_Ne: VocType_NoRe<br>CallType_So: VocType_NoRe<br>CallType_Te: VocType_NoRe<br>CallType_Th: VocType_NoRe<br>CallType_Wh: VocType_NoRe	-1.2642<br>0.23296<br>-0.29379<br>0.19397<br>0.18119<br>0.23806<br>-0.26702<br>0.49578<br>0.59811<br>-0.1853	0.14244<br>0.24279<br>0.32963<br>0.29181<br>0.35457<br>0.29091<br>0.34629<br>0.29075<br>0.28945<br>0.37088
CR ~ **VocRank** + VocType + (1|Subject) + (1|Subject:Date)	Text	42274	193	401.08	1	0	Intercept<br>VocRank<br>VocType	-0.40703<br>0.0031653<br>1.5898	0.13113<br>0.00015928<br>0.032316
CR ~ **RendRank** + VocType + (1|Subject) + (1|Subject:Date)<br>(Predictions of previous model set as an offset)	Text	42274	193	3.1644	1	0.075258	Intercept<br>RendRank<br>VocType	0.019391<br>-<br>0.00064723<br>-0.018183	0.01553<br>0.00036376<br>0.030157
**All-call-type tests data (n=13 subjects, with training on vocalizers)**<br>Int ~ **VocType** + (1|Subject)	6B	815	13	397.22	1	0	Intercept<br>VocType_NoRe	-1.2931<br>-1.3662	0.21406<br>0.072153
Int ~ VocType + **VocType:CallType** + (1|Subject)	6B	815	13	247.7	6	0	Intercept<br>VocType_NoRe<br>CallType_DC: VocType_NoRe<br>CallType_Ne: VocType_NoRe<br>CallType_So: VocType_NoRe<br>CallType_Te: VocType_NoRe<br>CallType_Th: VocType_NoRe<br>CallType_Wh: VocType_NoRe	-1.3147<br>1.1037<br>0.17097<br>0.14006<br>-0.94897<br>0.89095<br>0.76231<br>0.23767	0.22516<br>0.098767<br>0.10236<br>0.10312<br>0.13793<br>0.10745<br>0.10724<br>0.12678
Int ~ VocType + **CallType** + VocType:CallType + (1|Subject)	SF3A	815	13	48.877	6	7.8904×10^{-9}	Intercept<br>VocType_NoRe<br>CallType_DC<br>CallType_Ne<br>CallType_So<br>CallType_Te	-1.329<br>1.118<br>-0.19197<br>0.29606<br>-1.1555<br>0.054378	0.26718<br>0.17367<br>0.22869<br>0.22163<br>0.37725<br>0.22105

							CallType_Th	0.6066	0.21545
							CallType_Wh	-0.94894	0.332
							CallType_DC: VocType_NoRe	0.36296	0.25042
							CallType_Ne: VocType_NoRe	-0.15645	0.24427
							CallType_So: VocType_NoRe	0.20479	0.40154
							CallType_Te: VocType_NoRe	0.83655	0.24562
							CallType_Th: VocType_NoRe	0.15618	0.24037
							CallType_Wh: VocType_NoRe	1.1863	0.35503
Int ~ VocType + VocType:CallType + **VocType:Session +** (1|Subject)	7A	815	13	63.997	2	1.2657x10^{-14}	Intercept	-1.3093	0.22206
							VocType_NoRe	0.67514	0.11432
							CallType_DC: VocType_NoRe	0.1844	0.10305
							CallType_Ne: VocType_NoRe	0.16352	0.10388
							CallType_So: VocType_NoRe	-0.9587	0.13892
							CallType_Te: VocType_NoRe	0.93155	0.10838
							CallType_Th: VocType_NoRe	0.78433	0.10807
							CallType_Wh: VocType_NoRe	0.24298	0.12789
							VocType_NoRe:Session_2	0.4465	0.080781
							VocType_NoRe:Session_3	0.63939	0.080743
Int ~ VocType + **VocType:Session +** (1|Subject)	7A	156	13	59.497	2	1.2035x10^{-13}	Intercept	-1.2295	0.18032
							VocType_NoRe	1.0315	0.087201
							VocType_NoRe:Session_2	0.3796	0.076757
							VocType_NoRe:Session_3	0.58022	0.075566
Int ~ VocType + VocType:CallType + VocType:Session + **VocType:CallType:Session +** (1|Subject)	7A	815	13	28.653	12	0.0044348	Intercept	-1.3087	0.22174
							VocType_NoRe	0.29071	0.18058
							VocType_NoRe:Session_2	0.86238	0.20654
							VocType_NoRe:Session_3	1.1971	0.20711
							CallType_DC: VocType_NoRe	0.24215	0.22443
							CallType_Ne: VocType_NoRe	0.60611	0.21924
							CallType_So: VocType_NoRe	-0.30128	0.29365
							CallType_Te: VocType_NoRe	1.4959	0.21989
							CallType_Th: VocType_NoRe	1.4046	0.22641
							CallType_Wh: VocType_NoRe	0.72188	0.26265
							VocType_NoRe:Session_2: CallType_DC	-0.052912	0.27821
							VocType_NoRe:Session_3: CallType_DC	-0.058464	0.28128
							VocType_NoRe:Session_2: CallType_Ne	-0.38152	0.27635
							VocType_NoRe:Session_3: CallType_Ne	-0.74945	0.27652

							VocType_NoRe:Session_2: CallType_So<br>VocType_NoRe:Session_3: CallType_So<br>VocType_NoRe:Session_2: CallType_Te<br>VocType_NoRe:Session_3 : CallType_Te<br>VocType_NoRe:Session_2 : CallType_Th<br>VocType_NoRe:Session_3 : CallType_Th<br>VocType_NoRe:Session_2 :<br>CallType_Wh<br>VocType_NoRe:Session_3 :<br>CallType_wh	-0.92959<br>-0.80911<br>-0.68132<br>-0.80679<br>-0.58142<br>-1.0145<br>-0.6722<br>-0.58513	0.38563<br>0.35918<br>0.28088<br>0.28319<br>0.28748<br>0.28338<br>0.33982<br>0.33072
Int ~ **VocType** + (1|Subject)<br>(Dataset restricted to Session1)	7A	251	13	60.097	1	8.9928×10^{-15}	Intercept<br>VocType_NoRe	-1.4663<br>1.1152	0.23236<br>0.15176
Int ~ **VocType** + (1|Subject)<br>(Dataset restricted to renditions heard once or twice)	7B	128	13	66.865	1	3.3307×10^{-16}	Intercept<br>VocType_NoRe	-1.2627<br>1.0006	0.21855<br>0.12486
Int ~ VocType + **VocType:CallType** + (1|Subject)<br>(Dataset restricted to renditions heard once or twice)	7B	506	13	52.276	6	1.6413×10^{-9}	Intercept<br>VocType_NoRe<br>CallType_DC: VocType_NoRe<br>CallType_Ne: VocType_NoRe<br>CallType_So: VocType_NoRe<br>CallType_Te: VocType_NoRe<br>CallType_Th: VocType_NoRe<br>CallType_Wh: VocType_NoRe	-1.2744<br>0.89838<br>-0.26211<br>0.14924<br>-1.6617<br>0.63807<br>0.51948<br>0.37173	0.22399<br>0.248<br>0.2888<br>0.28295<br>0.43166<br>0.28483<br>0.29318<br>0.37086
Int ~ VocType + **CallType** + VocType:CallType + (1|Subject)<br>(Dataset restricted to renditions heard once or twice)	SF3C	506	13	17.234	6	0.0084594	Intercept<br>VocType_NoRe<br>CallType_DC<br>CallType_Ne<br>CallType_So<br>CallType_Te<br>CallType_Th<br>CallType_Wh<br>CallType_DC: VocType_NoRe<br>CallType_Ne: VocType_NoRe<br>CallType_So: VocType_NoRe<br>CallType_Te: VocType_NoRe<br>CallType_Th: VocType_NoRe	-1.2668<br>0.89096<br>-0.26649<br>0.23201<br>-0.83011<br>0.030938<br>0.45651<br>-1.2525<br>0.0037342<br>-0.083601<br>-0.82963<br>0.60819<br>0.066011	0.34632<br>0.36175<br>0.36786<br>0.34997<br>0.50253<br>0.3625<br>0.35144<br>0.67759<br>0.46771<br>0.45109<br>0.66277<br>0.46057<br>0.45727

							CallType_Wh: VocType_NoRe	1.6245	0.77271
**All-call-type tests data (n=7 subjects, without training on vocalizers)**<br>Int ~ **VocType** + (1|Subject)	6D	485	7	54.998	1	1.2068×10^{-13}	Intercept<br>VocType_NoRe	-0.93058<br>0.66923	0.15579<br>0.092388
Int ~ VocType + **VocType:CallType** + (1|Subject)	6D	485	7	172.37	6	0	Intercept<br>VocType_NoRe<br>CallType_DC: VocType_NoRe<br>CallType_Ne: VocType_NoRe<br>CallType_So: VocType_NoRe<br>CallType_Te: VocType_NoRe<br>CallType_Th: VocType_NoRe<br>CallType_Wh: VocType_NoRe	-0.93482<br>0.21915<br>0.36879<br>0.38103<br>-1.0822<br>1.0262<br>0.98912<br>0.35412	0.16139<br>0.13246<br>0.13842<br>0.13945<br>0.22263<br>0.14098<br>0.13829<br>0.17713
Int ~ VocType + **CallType** + VocType:CallType + (1|Subject)	SF3B	485	7	45.176	6	4.319×10^{-8}	Intercept<br>VocType_NoRe<br>CallType_DC<br>CallType_Ne<br>CallType_So<br>CallType_Te<br>CallType_Th<br>CallType_Wh<br>CallType_DC: VocType_NoRe<br>CallType_Ne: VocType_NoRe<br>CallType_So: VocType_NoRe<br>CallType_Te: VocType_NoRe<br>CallType_Th: VocType_NoRe<br>CallType_Wh: VocType_NoRe	-1.3955<br>0.67953<br>-0.044564<br>0.71687<br>-1.0529<br>0.74681<br>1.2292<br>0.18348<br>0.41312<br>-0.33552<br>-0.032003<br>0.28006<br>-0.23891<br>0.17027	0.26767<br>0.25018<br>0.33617<br>0.29621<br>0.52358<br>0.30053<br>0.29568<br>0.37753<br>0.36362<br>0.32741<br>0.5689<br>0.33188<br>0.32624<br>0.41687
Int ~ VocType + VocType:CallType + **VocType:Session +** (1|Subject)	7C	485	7	20.072	2	4.3799×10^{-5}	Intercept<br>VocType_NoRe<br>CallType_DC: VocType_NoRe<br>CallType_Ne: VocType_NoRe<br>CallType_So: VocType_NoRe<br>CallType_Te: VocType_NoRe<br>CallType_Th: VocType_NoRe	-0.93978<br>-0.087382<br>0.37272<br>0.38316<br>-1.1011<br>1.0417<br>0.9946	0.16471<br>0.15049<br>0.13895<br>0.13998<br>0.22333<br>0.14156<br>0.13887

							CallType_Wh: VocType_NoRe<br>VocType_NoRe:Session_2<br>VocType_NoRe:Session_3	0.36443<br>0.37017<br>0.44433	0.17787<br>0.10534<br>0.10351
Int ~ VocType + **VocType:Session +** (1|Subject)	7C	84	7	17.035	2	0.00019996	Intercept<br>VocType_NoRe<br>VocType_NoRe:Session_2<br>VocType_NoRe:Session_3	-0.93473<br>0.39785<br>0.33898<br>0.39294	0.15846<br>0.11432<br>0.10208<br>0.10026
Int ~ VocType + VocType:CallType + VocType:Session + **VocType:CallType:Session +** (1|Subject)	7C	485	7	14.488	12	0.27062	Intercept<br>VocType_NoRe<br>VocType_NoRe:Session_2<br>VocType_NoRe:Session_3<br>CallType_DC: VocType_NoRe<br>CallType_Ne: VocType_NoRe<br>CallType_So: VocType_NoRe<br>CallType_Te: VocType_NoRe<br>CallType_Th: VocType_NoRe<br>CallType_Wh: VocType_NoRe<br>VocType_NoRe:Session_2: CallType_DC<br>VocType_NoRe:Session_3: CallType_DC<br>VocType_NoRe:Session_2: CallType_Ne<br>VocType_NoRe:Session_3: CallType_Ne<br>VocType_NoRe:Session_2: CallType_So<br>VocType_NoRe:Session_3: CallType_So<br>VocType_NoRe:Session_2: CallType_Te<br>VocType_NoRe:Session_3 : CallType_Te<br>VocType_NoRe:Session_2 : CallType_Th<br>VocType_NoRe:Session_3 : CallType_Th<br>VocType_NoRe:Session_2 :<br>CallType_Wh<br>VocType_NoRe:Session_3 :<br>CallType_Wh	-0.94104<br>0.15089<br>0.084156<br>0.10065<br>-0.2266<br>0.12784<br>-2.0054<br>0.92828<br>0.83269<br>0.20515<br>0.55032<br>1.0264<br>0.33287<br>0.3479<br>1.0167<br>1.1645<br>0.17532<br>0.1081<br>0.29839<br>0.12521<br>0.18722<br>0.22877	0.16467<br>0.21868<br>0.26759<br>0.25927<br>0.28313<br>0.27717<br>0.6335<br>0.27221<br>0.27298<br>0.34585<br>0.36693<br>0.3603<br>0.3676<br>0.35291<br>0.73047<br>0.7126<br>0.36276<br>0.35488<br>0.35861<br>0.35187<br>0.45708<br>0.44884
Int ~ **VocType** + (1|Subject)<br>Dataset restricted to renditions heard once or twice	7D	289	7	8.8606	1	0.002914	Intercept<br>VocType_NoRe	-1.0024<br>0.47431	0.18237<br>0.16021
Int ~ VocType + **VocType:CallType** + (1|Subject)	7D	289	7	43.13	6	1.0994×10^{-7}	Intercept<br>VocType_NoRe	-1.0169<br>0.21267	0.19645<br>0.33451

Dataset restricted to renditions heard once or twice							CallType_DC: VocType_NoRe	-0.30844	0.40763
							CallType_Ne: VocType_NoRe	0.48079	0.38796
							CallType_So: VocType_NoRe	-2.2698	0.7944
							CallType_Te: VocType_NoRe	0.89623	0.3827
							CallType_Th: VocType_NoRe	0.62795	0.37971
							CallType_Wh: VocType_NoRe	0.52021	0.5185
Int ~ VocType + **CallType** + VocType:CallType + (1|Subject) Dataset restricted to renditions heard once or twice	SF3D	289	7	17.171	6	0.0086757	Intercept	-1.1026	0.39224
							VocType_NoRe	0.28787	0.47459
							CallType_DC	-0.14412	0.49052
							CallType_Ne	0.11956	0.46239
							CallType_So	-1.4324	0.70744
							CallType_Te	0.053968	0.46622
							CallType_Th	0.86865	0.45647
							CallType_Wh	0.19868	0.55916
							CallType_DC: VocType_NoRe	-0.15739	0.63866
							CallType_Ne: VocType_NoRe	0.36779	0.60446
							CallType_So: VocType_NoRe	-0.84396	1.0634
							CallType_Te: VocType_NoRe	0.8498	0.60301
							CallType_Th: VocType_NoRe	-0.23604	0.59354
							CallType_Wh: VocType_NoRe	0.32269	0.76389

Supplementary Methods:

Subjects and housing conditions

Subjects used for the behavioral experiments were thirteen adult domestic zebra finches (*Taeniopygia guttata*; 7 females and 6 males) bred in our colony at the University of California, Berkeley. The vocalization databank used as stimuli for the behavioral experiments and for the acoustic analyses has been previously described (see (1)) and was obtained from acoustic recordings of another set of 45 birds (20 females, 23 males and two chicks of unknown sex).

Birds were maintained at a constant temperature of 22–24°C and with a 14:10 light-dark cycle. Before the beginning of experiments, birds were housed in groups of 6-12 birds in a mix-sex environment. Food and water were provided *ad libitum*, with salad and egg supplement given once a week. For the duration of the shaping and testing days and while not in the testing chamber, the subjects were housed individually or in pairs in the colony room and fasted: their food intake was fixed to 1.5g of mixed seeds for finches per individual and was given at the end of each day upon returning to the colony room. The weight of each subject was closely monitored daily so that it remained between 85 and 90% of the initial body weight.

Behavioral experiments: study of the discrimination of vocalizers by zebra finches.

Chamber apparatus and test procedure

Fasting birds were tested in a computer-assisted extra tall modular test chamber (Med Associates Inc., St Albans, VT 05478, USA; size: 30.5 cm x 24.1 cm x 29.2 cm) placed in a soundproof booth (Acoustic Systems, MSR West, Louisville, CO, USA; interior dimensions 76 cm x 61 cm x 49 cm). The panel of the test chamber consists in a keypad placed 20.5 cm from the cage bottom and accessible via a wooden perch, and a feeder hole placed under the keypad at the bottom of the cage. Mixed seeds for finches are made accessible to the subject through the feeder hole for 10s as a reward. Acoustic stimuli are broadcasted by the computer monitoring the chamber via an amplifier (Technics, Matsushita Electronices, SA-EX140, Osaka, Japan) and a loudspeaker (Bose Model 141, Framingham, MA, USA) placed at 20 cm from the test chamber. The sound level is calibrated on song recordings to match the natural peak intensity levels of 70 dB SPL at 10 cm. The behavior of the subject is further monitored using a webcam (Logitech) placed inside the soundproof booth.

Sound playbacks and various functions of the test chamber were controlled by a computer running a custom program (Matlab, Mathworks, Cambridge, MA, USA), that communicated to the test chamber through a simple DAQ card (Measurement Computing Corporation, Norton, MA 02766, USA). The control of the test chambers included illuminating the key-pad, recording pecking events at 10Hz sampling rate and activating the feeder. A test consisted in three sessions of 30 min each per day with a minimum inter-session rest period of 90 min. The illumination of the key-pad signaled to the bird that it was active. The code detected the beginning of each session (when the bird pecked the-key pad for the first time) and ended the session 30 min later. Each hit on the key-pad triggered the playback of a different 6s stimulus. Interruption occurred when the bird pecked while the computer played a 6s stimulus resulting in the immediate trigger of another stimulus.

Acoustic stimuli

Each acoustic stimulus consisted of a sequence of six or three band-pass filtered (0.25-12 kHz) vocalizations of the same vocalizer and of the same call type, randomly assigned within a 6s window. More precisely, for the longer Begging sequences and Songs, each stimulus consisted of sequences of 3 different renditions, while for the other call types (Distance call, Nest call, Tet call, Tuck call, Whine call, Wsst call and Long Tonal call) each stimulus consisted of 6 different renditions. Each stimulus started and ended with a rendition. The 5 or 2 intervals between renditions in a given stimulus were randomly drawn from a uniform distribution. Before each session, the computer was randomly constructing a minimum of 80 Re stimuli and 320 NoRe stimuli using a vocalization bank of 5-104 (37.7±1.4) different renditions per vocalizer and per call type (see Supplementary Table 2). A total of 3283 vocalizations were used for these experiments. Each of the 400 stimuli (e.i. sequence of six or three different renditions) was only played once during the session.

Shaping

Birds were shaped to use the operant chamber over a short period of time (2-5 days) using two songs from different male zebra finches as Re and NoRe stimuli. Shaping consisted of the following steps: acclimation to the cage, finding the feeder, getting the association between pecking the key-pad and triggering a vocalization play-back and getting the association between hearing a Re vocalization and earning access to the feeder. Once the procedure to activate the feeder using the key-pad was acquired, birds were encouraged to interrupt by introducing the NoRe vocalization and increasing its probability in steps up to 80%. A subject was considered to have learned the task if it was pecking at least 50 times per day, interrupting the NoRe stimuli at least 20% of the time and if the percentage of interruption of NoRe stimuli was at least 20% higher than the percentage of interruption of Re stimuli.

Testing

For every subject, tests started on Day 0 with 3 sessions of discrimination between the two songs used during the shaping procedure. This way, each subject started the series of tests with the same prior experience with the apparatus, and having only heard stimuli that were different from those used in the actual experiment.

For each subject, a random pair of males, a random pair of females and a random pair of chicks were chosen from 24 vocalizers of our vocalization bank (7 females, 6 males and 11 chicks). Subjects were then tested for their ability to discriminate these vocalizers across all call types using the following 6 different types of discrimination tasks (Supplementary Table 2):

Male vocalizer single-call-type discrimination (7 tests per subject): discrimination of 2 male vocalizers within the same call type (7 call types each tested on consecutive days: Distance calls, DC-M; Nest calls, Ne-M; Songs, So-M; Tet calls, Te-M; Thuk calls, Th-M; Whine calls, Wh-M; and Wsst calls, Ws-M). For a given test (e.g. Te-M), all acoustic stimuli were constructed by randomly selecting and combining renditions of the same call type and emitted by the same individual (e.g., a Re stimulus consisted in 6 renditions chosen from 30 renditions of Tet calls from the same Re male).

Male vocalizer all-call-type discrimination (1 test per subject, All-M)*:* discrimination of 2 male vocalizers across all call types. In this test, each of the 7 adult call types was represented by 60 (NoRe vocalizer) and 12 (Re vocalizer) stimuli. The categories tested were: Distance calls, Nest calls, Songs, Tet calls, Thuk calls, Whine calls, and Wsst calls. Similar to the *vocalizer*

single-call-type discrimination task, each stimulus was constructed by randomly selecting and combining renditions of the same call type and emitted by the same individual.

Female vocalizer single-call-type discrimination (6 tests per subject): discrimination of 2 female vocalizers within the same call type (same call types as with male vocalizations with the omission of the Song that is not emitted by females, each tested on consecutive days: Distance calls, DC-F; Nest calls, Ne-F; Tet calls, Te-F; Thuk calls, Th-F; Whine calls, Wh-F; and Wsst calls, Ws-F). Acoustic stimuli were constructed following the same procedure as in *Male vocalizer single-call-type discrimination.*

Female vocalizer all-call-type discrimination (1 test per subject, All-F)*:* discrimination of 2 female vocalizers across all call types. In this test, each of the 6 female adult call types was represented by 54 (NoRe vocalizer) and 14 (Re vocalizer) stimuli. The categories tested were: Distance calls, Nest calls, Songs, Tet calls, Tuck calls, Whine calls, and Wsst calls. Similar to the *vocalizer single-call-type discrimination* task, each stimulus was constructed by randomly selecting and combining renditions of the same call type and emitted by the same individual.

Young vocalizer single-call-type discrimination (2 tests per subject): discrimination of 2 young vocalizers (chicks or C) within the same call type (2 call types each tested on consecutive days: Long Tonal call, LT-C and Begging calls, Be-C). Acoustic stimuli were constructed following the same procedure as in *Male vocalizer single-call-type discrimination.*

Random test (1 test per subject, RAN): Acoustic stimuli from two vocalizers of the same sex were prepared as for a *Vocalizer all-call-type discrimination* test but stimuli were randomly assigned to either the Re stimulus category or the NoRe stimulus category.

Note that *vocalizer single-call-type discrimination* tests were always performed before *vocalizer all-call-type discrimination* tests. For the *vocalizer single-call-type discrimination* tests, the order in which call types were tested was randomly assigned to each subject. Some tests were removed from the dataset because of stimulus assignment errors (Supplementary Table 2). All tests were performed in series of maximum 10 consecutive days and always started after a shaping day (Day 0).

To investigate the effect of the familiarity with vocalizations acquired during *vocalizer single-call-type discrimination* tests on the behavioral performance of birds during *vocalizer all-call-type discrimination* tests, 7 female subjects run an additional set of *vocalizer all-call-type discrimination* tests (All-F2 and All-M2) on vocalizations of birds they had never heard before.

Tests	# Males	# Females	# Re voc.	# NoRe voc.	Silence (s)	# voc. per stim
DC-M	6	7	22.5 +/- 2.7	22.8 +/- 2.5	4.733 +/- 0.002	6
Ne-M	6	6	36.4 +/- 6.3	40.2 +/- 5.7	4.712 +/- 0.006	6
So-M	6	7	19.2 +/- 3.9	18.6 +/- 4	0.693 +/- 0.006	3
Te-M	6	7	22.8 +/- 3.4	21.9 +/- 3.4	5.392 +/- 0.002	6
Th-M	6	7	17.8 +/- 4	16.2 +/- 3.5	5.581 +/- 0.001	6

Wh-M	6	7	18.8 +/- 3.7	20.2 +/- 3.8	4.317 +/- 0.006	6
Ws-M	6	7	19.8 +/- 3.6	20.5 +/- 3.8	3.831 +/- 0.007	6
All-M	6	7	116.4 +/- 17	129.2 +/- 20	4.122 +/- 0.014	3 or 6
All-M2	0	7	107.4 +/- 26.6	122.3 +/- 31.5	4.113 +/- 0.019	3 or 6
DC-F	6	7	30.6 +/- 3.4	28.8 +/- 3.7	4.647 +/- 0.002	6
Ne-F	6	7	20.3 +/- 4	20.8 +/- 4	4.251 +/- 0.007	6
Te-F	6	7	27.5 +/- 2.3	26.6 +/- 2.4	5.352 +/- 0.001	6
Th-F	6	7	31.6 +/- 4.8	30.1 +/- 5.1	5.6 +/- 0.001	6
Wh-F	6	2	4.8 +/- 1.4	4.8 +/- 1.4	2.545 +/- 0.007	6
Ws-F	6	7	16.7 +/- 2.9	14.4 +/- 2.9	3.942 +/- 0.006	6
All-F	6	7	115.3 +/- 13.2	117.7 +/- 17.7	4.727 +/- 0.007	6
All-F2	0	7	119.4 +/- 19.3	114.7 +/- 28.5	4.735 +/- 0.009	6
Be-C	6	7	18.8 +/- 1.3	19.5 +/- 1.3	1.567 +/- 0.007	3
LT-C	6	7	24.1 +/- 1.7	21.3 +/- 1.8	4.647 +/- 0.002	6
Random	6	6	240.4 +/- 28.6	240.4 +/- 28.6	4.409 +/- 0.012	3 or 6

Supplementary Table 2: Contingency table of the tests (n= 241 tests). The first two columns indicate the number of subjects that run each discrimination task. The third and fourth columns indicate the average number of vocalization renditions used to construct the Re stimuli and the NoRe stimuli. The fifth and sixth column gives respectively the average sum of silence periods and the number of vocalizations in a given 6 second stimulus.

Statistical analysis

Subjects performance at the discrimination task was measured by calculating the Odds Ratio (OR): the ratio between the odds of interrupting the NoRe vocalizer and the odds of interrupting the Re vocalizer.

$$OR = \log_2\left(\frac{O_{NoRe}}{O_{Re}}\right) = \log_2\left(\frac{p_{NoRe}/(1-p_{NoRe})}{p_{Re}/(1-p_{Re})}\right)$$

Here O_{NoRe} is the odds of interrupting a NoRe stimulus, O_{Re}, the odds of interrupting a Re stimulus, p_{NoRe}, the probability of interrupting a NoRe stimulus and p_{Re}, the probability of interrupting a Re stimulus. A time-varying OR was calculated by binning the data into windows that contained a fixed count of 4 Re stimuli and a varying count of NoRe stimuli depending on the contingent stimulus presentation (shown as the blue curve in Fig. 1B, 3C, 6A and 6C). An

overall OR was also calculated for each test by estimating probabilities using all the trials (shown as a large diamond marker placed on the right of the time-lines in Fig. 1B, 3C, 6A and 6C). To correct for the biases due to small numbers, the median unbiased estimate as proposed by Parzen et al. (2) were used for the calculation of probabilities of interruption p_{NoRe} and p_{Re}.

For a given test, the significance of the overall OR being different than 1 was calculated using an exact test: its value was compared to the distribution of OR values expected from two binomial distributions for the Re and NoRe interruptions, each with the corresponding observed number of trials, and assuming $p_{Re} = p_{NoRe} = 0.5$. A given value of OR was called significant if it was found in the upper percentile of the random distribution ($p < 0.01$).

Using the *glmefit* function of matlab, the behavioral performance across subjects was statistically tested with binomial Generalized Linear Mixed Effects models (GLME) where the response variable is the probability of interruption (Int) and the random variable is the bird subject (Subject). Birds are able to perform the task when models that include the Vocalizer Type (VocType) that codes whether a stimulus is from the Rewarded (Re) or Non-Rewarded (NoRe) vocalizer perform significantly better than models that don't include VocType (significance tested with a Likelihood ratio test, LRT) and the NoRe beta coefficient is positive. To investigate the effects of the call type (CallType) and of the session (Session) on the probability of stimulus interruption, these variables were added as co-variates in the previous model, and the comparison of models with or without the co-variate was conducted with an LRT to determine it significance. The subjects ID was set as a random variable in all these models. To test if the subjects were memorizing each rendition or generalizing across renditions to do the discrimination, we calculated, for each daily test, the rank of renditions (RendRank) and rank of vocalizer type (Re vs NoRe) presentation (VocRank). In the binomial GLME, the response variable considered was the probability of correct response (CR, probability of interrupting the NoRe or refraining from interrupting the Re), and the random variables were Subject and the day of the test (Date) nested within Subject. The response variable was changed here to maintain the power of the test despite the increase in the number of random groups in the GLME. Because VocRank and RendRank were highly correlated, the effect of RendRank was revealed by measuring its predictive power on CR once the effect of VocRank was removed. This was achieved by comparing two GLME with and without RendRank as a variable, but both predicting CR, with VocType as a co-variate, Subject and Date:Subject as random variables, and an offset based on the predictions p of a third GLME. This third GLME was predicting CR with VocType and VocRank as variables, Subject and Date:Subject as random variables. The offset was calculated as $\log(p/(1-p))$.

A fine description of all the GLME tests performed is given by Supplementary Table 1.

Acoustical analysis: study of the discrimination of vocalizers by classifiers.

Feature Spaces

The PAF (predefined acoustic features) consisted of 18 features describing the spectral (8), temporal (5) and fundamental (5) characteristics of each sound (see also (1)). The spectral features were extracted from the frequency power spectrum (called spectral envelope here). The spectral envelope was estimated using Welch's average periodogram (window = 49 ms, 50% overlap, Hanning window). From the normalized spectral envelope (to have unit integral), we calculated the first moments: the mean spectrum, the spectral standard deviation (i.e. the spectral bandwidth), the spectral skew and the spectral kurtosis. To capture an overall measure of spectral

envelope variability, we also calculated the spectral entropy. Finally, we also calculated the 3 quartiles (the 25% quartile, the median and 75% quartile) as these are often used in bioacoustical analyses. A temporal envelope was estimated by rectifying the sound pressure waveform and low-pass filtering at 20 Hz. From the normalized temporal envelope, we obtained the temporal mean, the temporal standard deviation (i.e. the duration), the temporal skew and temporal kurtosis. Overall variability was quantified with the temporal entropy. Five fundamental parameters were obtained from a time-varying estimation of the instantaneous fundamental frequency (1 kHz sampling). The fundamental (F0) was estimated using a hybrid temporal/spectral approach: the auto-correlation function of the signal was first analyzed to estimate the period of F0 based on the largest non-zero time-lagged peak in the auto-correlation function with a frequency below 1500Hz; this initial estimate was then used as an initial guess for matching the spectral periodicity found in the spectrogram at the corresponding time window (see Elie and Theunissen, 2016, for more details). The ratio of amplitude of the non-zero delay peak in the auto-correlation function with the peak at zero delay was used to estimate the periodicity of the sound at each time point. The pitch saliency of each vocalization was taken as the average value of this amplitude ratio over time points. F0 was only estimated for periodic time points showing values of pitch saliency above 0.5. In addition, we obtained the mean F0, the min F0, the max F0, and the coefficient of variation of F0. Equations and additional details for the calculations of PAF can be found in Elie and Theunissen (2016). Note that in this analysis, we did not use any features that described the intensity of the sound (e.g. RMS, peak amplitude) because these might have been affected by systematic differences in the position of the birds relative to the microphone and could bias the classifier for discriminating vocalizer identity. In some of our analyses, we used only the 8 spectral or only the 5 temporal or only the 5 fundamental features in the classifiers in order to compare the relative importance of these three types of acoustic features.

In addition to the PAF, we also used a practically invertible spectrographic representation to describe the sounds (3). The spectrogram was estimated using Gaussian-shaped windows (52 Hz wide in spectral domain and, correspondingly, 3 ms in the time domain) and resulted in 231 frequency bands between 0 and 12 kHz and a sampling rate of 1017 Hz yielding 357 points in time for the 350ms window used to frame each vocalization. In this representation, the vocalizations were centered within this 350ms window based on the time of the peak of their amplitude envelopes. Vocalizations for which the beginning or end occurred before the end of this spectrographic window were padded with zeros. Vocalizations that were longer than this time interval where truncated. In this manner, all sounds could be represented by the same 357x231= 82,467 feature vector. Similar to the PAF, we did not want to take into account the amplitude of the sound signal as an indicator of vocalizer identity. Thus, we normalized all spectrograms relative to their maximum amplitude.

Acoustic features described here were obtained using custom Python code from the Theunissen lab (BioSound class in sound.py found in https://github/theunissenlab/soundsig; BioSound Tutorials with examples are found in https://github/theunissenlab/BioSoundTutorials).

Classifiers

The three supervised classifiers (Linear discriminant analysis or LDA, quadratic discriminant analysis or QDA and random forest or RF) were used on all the data and with the same regularization procedure. Before training each classifier, principal component analysis was applied to the feature space chosen for sound representation (PAF or spectrograms) in order to

minimize over-fitting. In previous work (Elie and Theunissen, 2016), we systematically varied the number of principal components or PCs and chose the number that gave the best performance in cross-validated data. Here to minimize computational time and to use the same dimensionality reduction for all three classifiers, we used a prescriptive rule: the number of PCs used was equal to the square root of n/5, where n is the number of sounds used to train the classifier (this corresponds to approximately 10 degrees of freedom for each entry in the feature space covariance matrix). This dimensionality reduction step allowed us to have robust estimates of the stimulus covariance matrix.

The classification performance from regularized LDA, QDA and RF were very similar (see Supplementary Fig. 1) and results from LDA only are reported in the main paper. The classifiers software was based on the *scikit learn* library (version 0.19.1) for Python 2.7 (http://scikit-learn.org/stable/) augmented with the dimensionality reduction and cross-validation algorithms implemented in Theunissen Lab code (discriminate.py found in https://github.com/theunissenlab/soundsig ; tutorials in https://github.com/theunissenlab/BiosoundTutorial).

For the analyses on vocalizer discriminability, we chose a pair-wise approach where classifiers were trained and tested on all possible pair-wise comparisons of vocalizers. The pair-wise approach will be useful to compare the performance described in this paper with future work (in this species or other) that investigates vocalizer id or voice id and where the number of vocalizers tested or measured will vary. Indeed, we suggest that the methodology proposed here be used as a standard approach to study individual recognition such that comparative studies or meta analyses are facilitated.

Statistical analysis

The significance of the classifier performance for a given pair of vocalizers against chance (50%) was tested by an exact binomial test based on the number of vocalizations correctly classified and the total of number of vocalizations tested in the cross-validation procedure. Discrimination for a bird pair was considered significant if $p<0.05$. We then performed a second exact binomial test to determine whether the number of significant bird pair discriminations for a particular call type was above the 5% (expected Type 1 Error).

To obtain the average performance across all bird pair comparisons and for all call types, we fitted Generalized Linear Mixed Effects models (GLME) where the response variable is the number of cross-validated trials correctly classified versus total number tested, the fixed effect is the call type (CallType), the distribution is set to binomial and the random effect is the pair of vocalizers. The model coefficients of these GLME's are then used as the average responses and plotted on summary plots (such as in Fig. 4B). Furthermore, the effect of call type (CallType), training set (TrainSet) or the set of acoustic features used (Feature Space) were tested for significance by Likelihood ratio tests that compare the reduction in Deviance in models with, as compared to without, theses explanatory variables of interest to the expected reduction in Deviance that would be obtained by chance (Chi-Square test). GLME were fitted in R with the *lme4* library. The model coefficients and their 95% confidence intervals were obtained with the R *effects* library. A fine description of all the GLME tests performed is given by Supplementary Table 1.

Supplementary References

1. Elie JE & Theunissen FE (2016) The vocal repertoire of the domesticated zebra finch: a data-driven approach to decipher the information-bearing acoustic features of communication signals. *Anim Cogn* 19(2):285-315.
2. Parzen M, Lipsitz S, Ibrahim J, & Klar N (2002) An Estimate of the Odds Ratio That Always Exists. *Journal of Computational and Graphical Statistics* 11(2):420-436.
3. Singh NC & Theunissen FE (2003) Modulation spectra of natural sounds and ethological theories of auditory processing. *J Acoust Soc Am* 114(6 Pt 1):3394-3411.
