# Supplementary material for: Zebra finches identify individuals using vocal signatures unique to each call type
Source: Nat Commun. 2018 Oct 2;9:4026. doi: 10.1038/s41467-018-06394-9 (PMC6168511; doi:10.1038/s41467-018-06394-9)
Supplement: Supplementary file 3 — Description of Additional Supplementary Files [file 41467_2018_6394_MOESM3_ESM.pdf]

Description of Additional Supplementary Files:

Supplementary Movie 1. Female subject performing the single-call-type test on Distance calls from 2 males.

Supplementary Audio 1. **Wsst calls from Bird 1 and Bird 2.** The first 3 renditions are from Bird 1 and the last 3 from Bird 2.

Supplementary Audio 2. **Distance calls from Bird 1 and Bird 2.** The first 3 renditions are from Bird 1 and the last 3 from Bird 2.
